# Supplementary material for: Loss of Nckx3 Exacerbates Experimental DSS-Induced Colitis in Mice through p53/NF-κB Pathway
Source: Int J Mol Sci. 2021 Mar 5;22(5):2645. doi: 10.3390/ijms22052645 (PMC7961925; doi:10.3390/ijms22052645)
Supplement: Supplementary file 1 [file ijms-22-02645-s001.pdf]

Supplementary Table S1. Primers used for gene expression analysis through Real-Time PCR.

	Forward	Reverse
Ifit1	GGCCGTTTCCTACAGTTTCA	AGCCCAAGAAGGCTGGTACT
Zbp1	TGACGACAGCCAAAGAAGTG	TGTGGGTTCAAGACCAGCTT
Irf7	CCCAAGGAGAAGACCCTGAT	CCAGGTCCATGAGGAAGTGT
Oasl1	ATTGCACGCTCGCCTACTAC	AGGCCCTGCCTGTCTTCTAT
Oasl2	ACAGCCCCATCAGAAAGATG	GGAAAGGTCTCCAGCACTTG
Erdr1	ACAGTGATGTCACCCACGAA	GGCATTTCTGTACGCAGTCA
Stat1	GTGGAGCCCTACACGAAAAA	TCTGGTGCTTCCTTTGGTCT
Irgm2	CGCGATAGAGATTCGGAAAG	GGGTTCCAGTGCGATACAAC
Anpep	GTGAACGAAGCGGACAAACT	GGACAAAGTCCCAAACCAGA
Rtp4	CAAAGAGTTCCTCCCCATCA	GGCAAATGCAGCAATAGACA
Isg15	GGCCACAGCAACATCTATGA	CGCAAATGCTTGATCACTGT
Usp18	GATTCTCTGCCAGGAACTCG	ATGACCAAAGTCAGCCATCC
Dnase1	TGCAGCCTTCAACATTCGGA	GGTGTCAGGTTTGTCCCGAT
Gkn1	CCTGGTTTTGCTTACACGGTC	GGAGTCCCAGCCGTTATTGT
Gkn2	ACTGGTTCCTGTTTGGGTCG	GCTCCAGTGCTCACCTCTTT
Gkn3	TACCCAGCCGAATCAAAAAC	AGGGATCTCACCACAAATGG
Tff1	CAGGCCCAGGAAGAAACAT	TCTTCTTGAGTGTTCTCGATGG
Tff2	GTTTCCACCCACTTCCAAAC	CAGACTGTGGGAAGAAACACC
Grem1	TTCTGTTATGGCCAGTGCAA	GCGTGTGACCCTTTTCTTCT
Anxa10	TGGATGCCATCAATGACTGT	TTTCACTTCTGGCGATGAGA
Acta2	AGCCATCTTTCATTGGGATG	AGGGCTGTGATCTCCTTCTG
Agr2	AAGCACCTTTCTCCTGATGG	GCAGCTTGAGAGCTTTCTTCA
Clu	CGAAGATGCTCAACACCTCA	AACAGCTTCACCACCACCTC
Reg3g	CAACAGAGGTGGATGGGAGT	GGCCTTGAATTTGCAGACAT
Fxyd3	GGGCTCATTTGTGCAGGGAT	AGCTGAGCCTGGTGTGATGA

Mt2	GCCTGCAAATGCAAACAATG	CACTTGTCGGAAGCCTCTTT
Muc5ac	CTGCTTCTGTCCTGAGGGTATG	CATGTGTTGGTGCAGTCAGTAG
Muc6	GCCAACCAAGTGTACCAGGA	ATCAAGCAGTGTACCGTGGG
TNF	CCTGTAGCCCACGTCGTAG	GGGAGTAGACAAGGTACAACCC
IFN-a	TGCAGGAATTTCCCCTGACC	AAGACAGGGCTCTCCAGACT
IFN-b	AACTCCACCAGCAGACAGTG	CATCCAGGCGTAGCTGTTGT
IL-1a	TCGGGAGGAGACGACTCTAAAT	GTTTCTGGCAACTCCTTCAGC
IL-1b	TGCCACCTTTTGACAGTGATG	TGATACTGCCTGCCTGAAGC
IL-6	GTTCCTCTCTGCAAGAGACTTC	TGGGAGTGGTATCCTCTGTG
p53	TGGAGGAGTCACAGTCGGAT	CAGTGAGGTGATGGCAGGAT
p65	CTGCCGAGTAAACCGGAACT	GCCTGGTCCCGTGAAATACA
